# Poor patient-reported outcome after shoulder replacement in young patients with cuff-tear arthropathy: a matched-pair analysis from the Danish Shoulder Arthroplasty Registry

**DOI:** 10.1080/17453674.2018.1563855

**Published:** 2019-01-23

**Authors:** Mette Ammitzboell, Amin Baram, Stig Brorson, Bo Sanderhoff Olsen, Jeppe V Rasmussen

**Affiliations:** a Department of Orthopaedic Surgery, Herlev and Gentofte Hospital, University of Copenhagen;; b Department of Orthopaedic Surgery, Zealand University Hospital, University of Copenhagen, Denmark

## Abstract

Background and purpose — Reverse shoulder arthroplasty (RSA) has become the treatment of choice for cuff-tear arthropathy. There are, however, concerns about the longevity and the outcome of an eventual revision procedure. Thus, resurfacing hemiarthroplasty (RHA) with extended articular surface has been suggested for younger patients. We compared the patient-reported outcome of these arthroplasty designs for cuff-tear arthropathy.

Patients and methods — We included patients operated on because of cuff-tear arthropathy and reported to the Danish Shoulder Arthroplasty Registry (DSR) from January 1, 2006 to December 31, 2013. 117 RHA cases were matched by age and sex with 233 RSA controls. 34 of the RHAs were conventional and 67 were RHAs with extended articular surface. The Western Ontario Osteoarthritis of the Shoulder (WOOS) Index at 1 year was used as primary outcome. The score was converted to a percentage of a maximum score. Revision, defined as removal or exchange of any component or the addition of a glenoid component, was used as secondary outcome.

Results — Median WOOS was 49 (30–81) for RHA and 77 (50–92) for RSA (p < 0.001). For patients younger than 65 years, median WOOS was 58 (44–80) after RHA, similar to the 54 after RSA (37–85). For patients older than 65 years, median WOOS was 48 (28–82) after RHA and 79 (55–92) after RSA (p < 0.001).

Interpretation — In all patients RSA had a clinically and statistically better patient-reported outcome than RHA. However, in patients younger than 65 years the functional outcome was similar and poor for either arthroplasty type. The optimal treatment of CTA in young patients remains a challenge.

According to Neer et al. (1983), cuff-tear arthropathy (CTA) is described as a large rotator cuff tear followed by inactivity, disuse of the shoulder, leaking of synovial fluid, and instability of the humeral head. Radiographic characteristics are superior migration, collapse of the proximal aspect of the humeral articular surface, and erosion or acetabularization of the acromion (Neer et al. 1983, Feeley et al. 2009). The clinical symptoms are pain, arthritis, muscle atrophy, decreased range of motion, and in a subset of late-stage patients a pseudoparalytic shoulder (Neer et al. 1983, Ramirez et al. 2012, Smith et al. 2012). In these cases, the result of reverse shoulder arthroplasty (RSA) is better than that of resurfacing hemiarthroplasty (RHA) (Young et al. 2013). There are, however, concerns not only regarding the longevity of RSA but also the outcome of an eventual revision procedure. Thus, RHA with posterior and superior extended articular surface has been suggested as an option in the treatment of younger patients with long life expectancy.

We compared patient-reported outcome and revision rates of RHA and RSA in the treatment of CTA in average patients and in patients younger than 65 years.

## Patients and methods

Data were obtained from the Danish Shoulder Arthroplasty Registry (DSR). All Danish public hospitals and private clinics report to this registry. More than 90% of the procedures have been captured each year since 2007 when compared with the national patient registry (DSR 2016). Surgeons report information concerning the patient (name, date of birth, and sex) and the procedure (hospital, date of surgery, diagnosis, previous surgery, and arthroplasty type) electronically at the time of the operation. 10–14 months after the procedure, the patient-reported outcome is collected using the Western Ontario Osteoarthritis of the Shoulder (WOOS) questionnaire index. WOOS consists of 19 questions focused on shoulder-related quality of life. Each question is answered on a scale from 0 to 100; thus the total score ranges from 0 to 1,900. In this study, the raw WOOS score was converted to a percentage of a maximum score where a score of 100 was regarded as a healthy shoulder with no functional impairment (Lo et al. 2001). A validated Danish version of WOOS is used in the DSR (Rasmussen et al. 2013). To our knowledge, a minimal clinically important difference WOOS has not been established and validated. We used a value of 10% of a maximum score, which is an extrapolation from other shoulder-specific questionnaires (e.g., the Oxford Shoulder Score [OSS)]) (Rasmussen et al. 2013). We defined a clinical failure as a WOOS below 50. Revision is defined as removal or exchange of any component or the addition of a glenoid component.

All primary shoulder arthroplasties for CTA reported to the registry from January 1, 2006 to December 31, 2013 were identified (n = 891). We matched each RHA (n = 119) with 2 RSA controls based on age, sex, and response of the WOOS at 1 year. For patients younger than 60 years we accepted matches in intervals of 5 years. 2 patients did not have matching controls available (they were both men and 43 years old). 1 had a WOOS of 73 and 1 was recorded as a non-responder regarding WOOS. A 49-year-old man with a WOOS of 47 was matched with only 1 control. Thus, 117 RHAs and 233 RSAs were included in the study. 73% had a complete WOOS, 24% did not respond to WOOS, and 3% were revised or died within the first year after surgery.

### Statistics

Data on WOOS were not normally distributed. Thus, the results are presented as median and interquartile range (IQR) and the Mann–Whitney U-test was used when groups were compared. P-value < 0.05 was considered significant. The analysis was performed using IBM SPSS statistics version 24.0 (IBM Corp, Armonk, NY, USA)

### Ethics, funding, and potential conflicts of interest

Ethics approval was from the Danish Patient Safety Department (Study number 3-3013-1862/1/Reference MOAD from March 28, 2017). No funding was recieved. No conflicts of interest.

## Results

The mean age of all patients was 73 years (50–92) and 60% were women. The median WOOS for all patients was 49 (30–81) for RHA and 77 (50–92) for RSA, (p < 0.001). There were 44 (52%) RHAs and 42 (25%) RSAs with a WOOS below 50 (Figure). 7 (6%) RHAs and 13 (6%) RSAs were revised. The reasons for revision after RHA were glenoid attrition (n = 2), infection (n = 1), luxation (n = 1), other (n = 1), or no reason reported (n = 2). The reasons for revision after RSA were infection (n = 5), luxation (n = 3), loosening (n = 3), other (n = 1), or no reason reported (n = 1).

**Figure:**
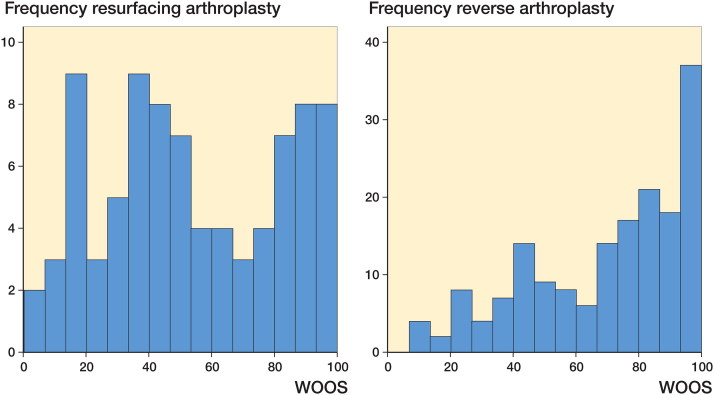
WOOS distribution after resurfacing arthroplasties (left panel) and reverse arthroplasties (right panel).

Of the 117 RHAs, 34 were conventional RHAs and 67 had posterior and superior extended articular surface. Information regarding the subtype was missing in 16 cases. In the group with posterior and superior extended articular surface 35 (52%) patients were women, mean age was 73 years, and the response rate of WOOS was 73%. In the group with conventional RHAs 22 (65%) patients were women, mean age was 73 years, and the response rate of WOOS was 71%. The median WOOS was 48 (33–80) for RHA with posterior and superior extended articular surface and 57 (38–92) for conventional RHA (p = 0.4). 5 (8%) RHAs with posterior and superior extended articular surface and 1 (3%) conventional RHA were revised.

For patients younger than 65 years the median WOOS was similar for RHA (n = 19) and RSA (n = 32) at 58 (44–80) and 54 (37–85). For patients older than 65 years the median WOOS for RHA (n = 66) and RSA (n = 137) was 48 (28–82) and 79 (55–92) (p < 0.001).

## Discussion

We found the patient-reported outcome after RSA to be better than after RHA. For patients younger than 65 years the results were disappointing for both arthroplasty types. The revision rates were the same. 52% of RHAs and 25% of RSAs had a WOOS below 50 but only 6% were revised for both arthroplasty designs. A reason for this difference might be restraint in revision arthroplasties because of poor outcome.

The results of RHA or RSA for CTA have frequently been reported in small case series, but to our knowledge there are only 2 studies comparing hemiarthroplasty with RSA for CTA. From the New Zealand Joint Registry Young et al. (2013) compared 102 hemiarthroplasties with 102 RSAs. 77 of 102 hemiarthroplasties were RHAs with posterior and superior extended articular surface. The patients were matched for age, sex, and ASA scores, and the functional outcome was evaluated using the OSS at 6 months and 5 years postoperatively (Young et al. 2013). They found no differences in mortality, revision rates, or OSS for patients younger than 65 years when comparing 24 hemiarthroplasties and 20 RSA. As in our study there was a statistically significant difference in functional outcome overall and in patients older than 65 years. The authors did not report separate results of the 77 RHAs with posterior and superior extended articular surface (Young et al. 2013). A retrospective study by Leung et al. (2012) from a 10-year period compared 20 hemiarthroplasties and 36 RSAs for CTA at minimum 2 years’ follow-up. They found a better Shoulder Pain and Disability Index (SPADI) for RSA. They found high complication rates in 14 patients from both arthroplasty types, with infections and pain in the hemiarthroplasty group and infection, loosening, and fracture of acromion or humerus in the RSA group (Leung et al. 2012).

A radiographic study by Leung et al. (2017) with 97 arthroplasties reported 26 radiographic complications, e.g., acromion remodeling, fracture, subluxation, or loosening after RHA with posterior and superior extended articular surface for CTA. Most of these complications were seen within the first 3 months postoperatively. Occurrence of postoperative radiographic complications was associated with revision. 8 arthroplasties were revised (Leung et al. 2017). No functional outcome or results, particularly for younger patients, were reported. Alizadehkhaiyat et al. (2013) compared RHA with posterior and superior extended articular surface with conventional RHA for different diagnoses including 9 patients with CTA of whom 8 received RHA with posterior and superior extended articular surface. 5 of these 8 patients were revised. The CTA group had a mean OSS of 26 postoperatively at 4 years’ follow up. The authors concluded that cuff deficiency was a major reason for failure and revision (Alizadehkhaiyat et al. 2013). Our results of conventional RHA and RHA with posterior and superior extended articular surface show poor patient- reported outcome for both types.

In a single-center retrospective study Ernstbrunner et al. (2017) looked at RSA for irreparable rotator cuff tears in 23 cases younger than 60 years. The functional and radiographic results at 10 years were good, but 6 patients were reoperated, mainly because of instability. The high revision rate suggests that RSA might not be the best choice for all younger patients. In the absence of alternative treatments a selected group of younger patients with irreparable rotator cuff tears can obtain good results with RSA (Ernstbrunner et al. 2017). To our knowledge there are no studies reporting the results of revision arthroplasty after failed RHA in young patients with CTA, but data from the DSR showed poor outcome of the revision arthroplasty when the RHA was used for osteoarthritis (Rasmussen et al. 2016). Consequently the use of shoulder arthroplasty for younger patients should be considered carefully.

The strength of our study is the systematic nationwide collection of data through the DSR with high external validity. There is no data selection, and our results reflect the actual outcome in the Danish population. The study has the limitations of observational studies including the possibility of differences in baseline characteristics such as in comorbidities and preoperative functional status. Another limitation of our study and other studies reporting the results of shoulder arthroplasty for CTA is the lack of a common definition and classification of CTA. There is a possible bias in distribution of surgeries among the participating centers and surgeons. We do not know whether indications for surgery or revisions are the same as in the studies used for comparison. Finally, we have no preoperative WOOS.

To conclude, RSA had a clinically and statistically significant better patient-reported outcome compared with RHA. However, for patients younger than 65 years the functional outcome was disappointing for both arthroplasty types. The optimal treatment of CTA in young patients remains a challenge.
